# Case Report: First Case of Cefotaxime-Sulbactam-Induced Acute Intravascular Hemolysis in a Newborn With ABO Blood Type Incompatibility by the Mechanism of Non-Immunologic Protein Adsorption

**DOI:** 10.3389/fimmu.2021.698541

**Published:** 2021-12-22

**Authors:** Yuanjun Wu, Yong Wu, Yong Yang, Baochan Chen, Jianqun Li, Ganping Guo, Fu Xiong

**Affiliations:** ^1^ Department of Blood Transfusion, Dongguan Maternal and Child Health Hospital, Dongguan, China; ^2^ Department of Blood Transfusion, Dongguan Tungwah Hospital, Dongguan, China; ^3^ Department of Neonatology, Dongguan Maternal and Child Health Hospital, Dongguan, China; ^4^ Department of Medical Genetics, School of Basic Medical Sciences, Southern Medical University, Guangzhou, China; ^5^ Key Laboratory of Single Cell Technology and Application in Guangdong, Guangzhou, China

**Keywords:** hemolytic disease of newborn (HDN), cefotaxime, sulbactam, drug-induced immune hemolytic anemia (DIIHA), non-immunologic protein adsorption (NIPA), acute intravascular hemolysis

## Abstract

**Background:**

ABO blood type incompatibility hemolytic disease of newborn (ABO-HDN) and drug-induced immune hemolytic anemia (DIIHA) due to non-immunologic protein adsorption (NIPA) mainly cause extravascular hemolysis. All the reported severe DIIHA were caused by drug-induced antibodies, and rare report of acute intravascular hemolysis was caused by the NIPA mechanism or ABO-HDN.

**Case presentation:**

We report the first case of acute intravascular hemolysis induced by cefotaxime sodium - sulbactam sodium (CTX - SBT) in a case of ABO-HDN which resulted in death at 55 h after birth. The mother’s blood type was O and RhD-positive, and the newborn’s blood type was B and RhD-positive. No irregular red blood cell (RBC) antibodies or drug-dependent antibodies related to CTX or SBT was detected in the mother’s plasma and the plasma or the RBC acid eluent of the newborn. Before the newborn received CTX - SBT treatment, the result of direct antiglobulin test (DAT) was negative while anti-B was positive (2 +) in both plasma and acid eluent. After the newborn received CTX - SBT treatment, the results of DAT for anti-IgG and anti-C3d were both positive, while anti-B was not detected in plasma, but stronger anti-B (3 +) was detected in acid eluent. *In vitro* experiments confirmed that NIPA of SBT promoted the specific binding of maternal-derived IgG anti-B to B antigen on RBCs of the newborn, thereby inducing acute intravascular hemolysis.

**Conclusion:**

The NIPA effect of SBT promoted the specific binding of mother-derived IgG anti-B in newborn’s plasma to the newborn’s RBC B antigens and formed an immune complex, and then activated complement, which led to acute intravascular hemolysis. Drugs such as SBT with NIPA effect should not be used for newborns with HDN.

## Introduction

Drug-induced immune hemolytic anemia (DIIHA) is a rare disease resulting from immune damage to red blood cells (RBCs) caused by drug-induced antibodies or non-immunologic protein adsorption (NIPA) (1–4), and nearly 140 drugs have been reported to cause DIIHA *via* drug-induced antibodies, and approximately 10 *via* NIPA (2–12). Cefotaxime (CTX) is a third-generation cephalosporin, which is a β-lactam broad-spectrum antibiotic. The antibacterial mechanism of CTX is mainly through inhibiting the cell wall penicillin binding proteins to hinder the synthesis of cell wall mucopeptides, causing the bacterial cell wall to be damaged and lysed. Because some pathogenic bacteria can produce β-lactamase and are resistant to CTX (13). Sulbactam (SBT) is an irreversible competitive β-lactamase inhibitor. The combined administration of CTX and SBT can avoid the resistance of pathogenic bacteria to CTX and improve the antibacterial effect (14). Cefotaxime sodium - sulbactam sodium (CTX-SBT) combined administration is commonly used in diverse bacterial infections (15). However, DIIHA caused by CTX and SBT has been reported in some cases (2, 16, 17), and DIIHA caused by CTX-induced drug-dependent antibodies can cause intravascular hemolysis although it is rare (16), while SBT can only through the NIPA mechanism cause positive direct anti-globulin tests (DAT) results and slow extravascular hemolysis that is difficult to observe (2, 3). To date there are no reports of SBT-induced drug-dependent antibodies and NIPA induced by CTX. In addition, hemolytic disease of newborn (HDN) is a type of extravascular hemolysis which is usually caused by mother–infant ABO incompatibility in Asians (18–22).

Here, we report the first case of acute intravascular hemolysis in a newborn with ABO blood type incompatibility hemolytic disease of newborn (ABO-HDN) induced by CTX-SBT, which did not caused by CTX-induced drug-dependent antibodies but the NIPA of the SBT. The mother’s blood type was O and RhD-positive, and the newborn’s blood type was B and RhD-positive. No irregular RBC antibodies or drug-dependent antibodies related to CTX or SBT were detected in the plasma or the RBC acid eluent of the newborn, nor in the plasma of the mother. Before the newborn received CTX-SBT treatment, the results of DAT for anti-IgG and anti-C3d were negative and anti-B was positive (2+) in both plasma and acid eluent. However, after the newborn received CTX-SBT treatment, the results of DAT for anti-IgG (3+) and anti-C3d (2+) were positive, and anti-B was not detected in plasma, while stronger anti-B (3+) was detected in acid eluent. Further investigation revealed that acute intravascular hemolysis was caused by a NIPA mechanism of SBT promoting specific binding of maternal IgG-B to the newborn’s RBC B antigens, which in turn activated complement. This is a new understanding of the mechanism with NIPA of drugs. This study shows that the administration of drugs with NIPA-effect such as SBT for newborns with incompatible mother–infant blood type has a great risk, and drugs with NIPA-effect should be avoided as possible to avoid aggravating hemolysis of newborns.

## Case Report

### Patient Information

A male newborn with blood type B and RhD-positive was delivered by cesarean section at 39 weeks due to “pregnancy with uterine scar” in a second-level hospital. His mother was 24 years old with blood type O and RhD-positive, and this was her third delivery. There was no history of special medication before delivery. The newborn’s birth weight was 3.2 kg, his Apgar scores were10-10-10 at 1, 5 and 10 min after birth, and there was no history of intrauterine distress. After birth, he was fed with breast milk and exhibited normal suckling, urine and feces. Twenty hours after birth, the newborn’s skin became yellowish, and the color gradually worsened. Thirty-two hours after birth, blood samples were collected and examined. Detailed examination results are shown in **Table 1**. The newborn was diagnosed with ABO-HDN, and was treated with light therapy and intravenous injection of human immunoglobulin.

**Table 1:** Blood indexes changes of the newborn before and after CTX-SBT treatment.

Detection items	At Birth	Before CTX-SBT treatment	5h CTX-SBT treatment	Reference Range
	32h	(At birth 43h)	(At birth 48h)	(0-7 days after birth)
Serum or plasma color	yellow	yellow	dark red	light yellow
*Blood cell and biochemical detection*				
G-6PD (U/L)	4580.90	NT	NT	2500.00-5800.00
Hemoglobin (g/L)	160.00	191.00	80.00	170.00-200.00
Hematocrit (%)	45.50	57.30	17.30	51.00-60.00
RBC count (×10^12^/L)	4.20	5.04	2.43	5.20-6.40
Mean corpuscular volume (fL)	108.30	113.70	71.20	80.00-100.00
Mean corpuscular hemoglobin (pg)	38.20	37.90	32.90	27.00-31.00
MCHC (g/L)	353.00	333.00	462.00	320.00-360.00
RDW (%)	15.90	16.10	32.40	6.00-15.00
Reticulocyte proportion (%)	4.50	NT	NT	3.00-6.00
Platelet count (×10^9^/L)	258.00	321.00	85.00	100.00-300.00
WBC count (×10^9^/L)	16.55	8.39	28.11	15.00-20.00
Total bilirubin (μmol/L)	269.00	NT	73.80	12.00-217.00
Direct bilirubin (μmol/L)	9.00	NT	5.00	0.00-10.00
Indirect bilirubin (μmol/L)	260.00	NT	68.80	0.00-180.00
ALT(U/L)	23.00	NT	96.70	1.00-25.00
GOT(U/L)	44.00	NT	1044.50	10.00-50.00
LDH(U/L)	478.00	NT	9266.00	145.00-765.00
CK(U/L)	883.00	NT	1623.00	18.00-198.00
CK-MB(U/L)	16.20	NT	1466.00	<12.00
Serum potassium (mmol/L)	4.71	NT	7.46	3.60-4.80
*Immunohematological detection*				
ABO	B	B	B	/
RhD	Positive	Positive	Positive	/
Plasma anti-A	3+	3+	2+	/
Plasma anti-B	2+	2+	Negative	/
Irregular red blood cell antibodies	Negative	Negative	Negative	Negative
Drug-dependent antibodies	Negative	Negative	Negative	Negative
Red blood cell acid eluent anti-B	2+	2+	3+	Negative
Direct anti-globulin test for anti-IgG	Negative	Negative	3+	Negative
Direct anti-globulin test for anti-C3d	Negative	Negative	2+	Negative

SBT, sulbactam; CTX, cefotaxime; G-6PD, Glucose 6-phosphate dehydrogenase; RBC, red blood cell; MCHC, mean red blood cell hemoglobin concentration; RDW, red cell volume distribution width; WBC, white blood cell; ALT, alanine transaminase; GOT, glutamic-oxalacetic transaminase; LDH, lactate dehydrogenase; CK, creatine kinase; CK-MB, myocardial creatine kinase; NT, no tested.

Forty three hours after birth, the newborn excreted 3mL of stool with mucus and blood, with shortness of breath and restless crying. Examination results are shown in **Table 1**. An abdominal X-ray showed accumulation of gas in the bowel, and the newborn was diagnosed with necrotizing enterocolitis. He was treated with intravenous injection of vitamin K1 and a solution of 50 mL 0.9% NaCl and 0.15 g CTX-SBT (Cefotaxime and Sulbactam, 2:1). One hour later, the newborn’s breathing became shallow and he was diagnosed with metabolic acidosis. He was given endotracheal intubation and breathing machine auxiliary ventilation, and immediately transferred to a tertiary hospital.

**Figure 1:**
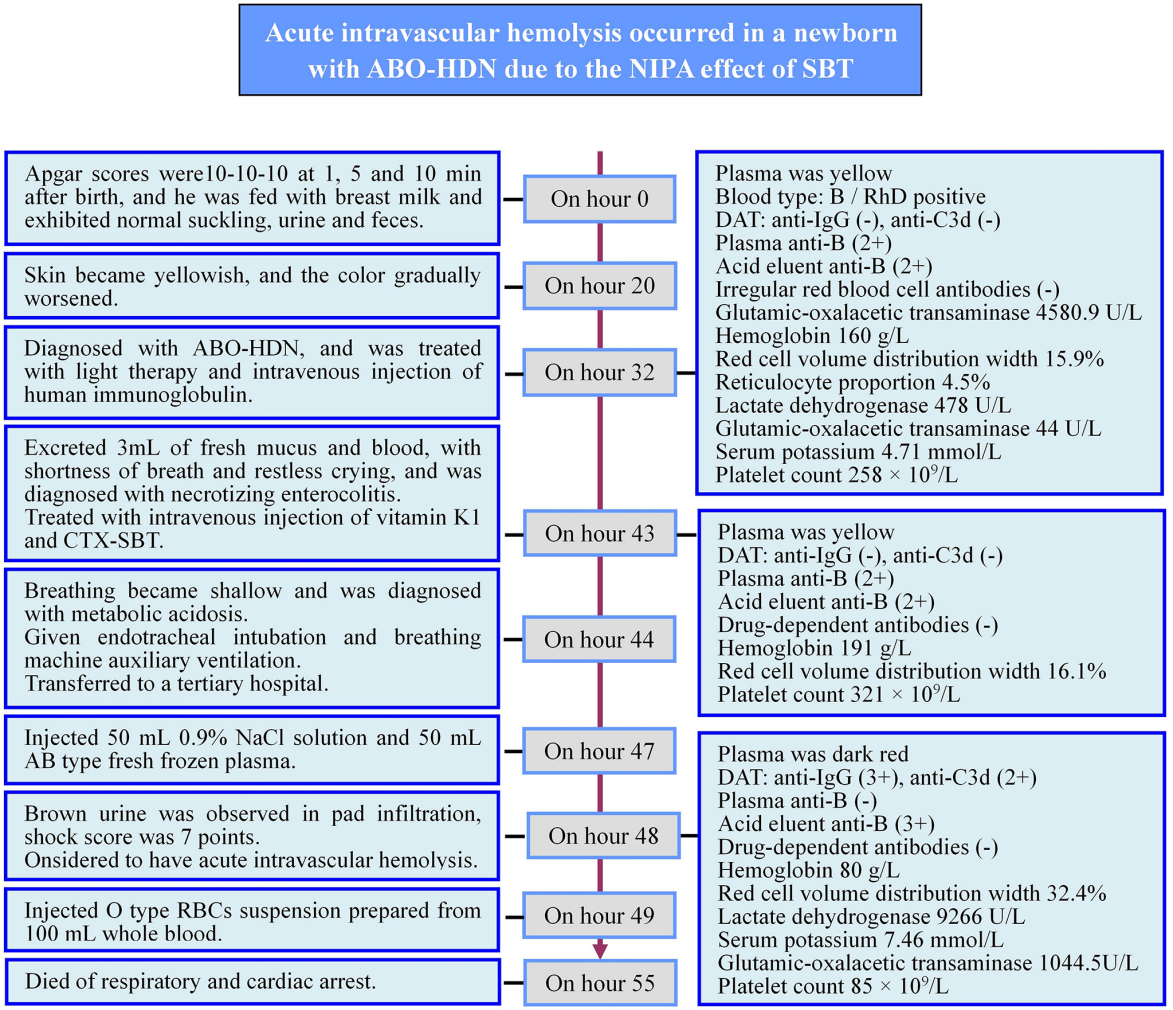
Timeline of the newborn disease process. ABO-HDN, ABO blood type incompatibility hemolytic disease of newborn; DAT, direct antiglobulin test; CTX-SBT, cefotaxime sodium - sulbactam sodium combination.

After hospital transfer, 50 mL 0.9% NaCl solution and 50 mL AB type fresh frozen plasma were injected to supplement the newborn’s blood volume. Five hours after the CTX-SBT treatment (48 h after birth), blood samples were examined and the results are shown in **Table 1**. Brown urine was observed in pad infiltration, the newborn’s blood pressure was 41/29 (31) mmHg, shock score was 7 points, and the metabolic acidosis was further aggravated. The newborn was considered to have acute intravascular hemolysis. Blood volume replenishment was continued, and he was injected with O type washed red blood cells (WRBCs) prepared from 100 mL whole blood. However, the newborn died of respiratory and cardiac arrest 12 h after the CTX-SBT treatment (55 h after birth). The 32, 43 and 48 hours after birth, blood samples were collected and examined, respectively. Detailed examination results are shown in **Table 1** and **Figure 1**.

### Serological Test Results

The ABO blood type and RhD of the mother and the newborn were identified. Blood samples collected at 32 h, 43 h (before CTX-SBT treatment) and 48 h (5 h after CTX-SBT treatment) of the newborn after birth were centrifuged to separate the RBCs, and the acid elution tests were performed after washing the RBCs four times. The micro-column gel antiglobulin method was used to screen irregular RBC antibodies in the blood samples of the mother and the newborn before CTX-SBT treatment. The micro-column gel antiglobulin method was also used to detect the titers of IgG ABO blood group antibodies in the mother’s plasma, and was also used to detect the ABO blood group antibodies in the plasma and the acid eluent of the blood samples collected before and after the newborn received CTX-SBT treatment. The blood samples collected at 32 h, 43 h (before CTX-SBT treatment) and 48 h (5 h after CTX-SBT treatment) of the newborn after birth, and the blood samples of the mother were performed DAT using mono-specific anti-IgG and anti-C3d. According to references (23), the “testing drug-induced antibodies with drug-treated RBCs” and the “testing drug-induced antibodies in the presence of a drug solution” were used to detected whether there were drug-induced antibodies related to CTX or SBT in the mother’s plasma, and in plasma or the acid eluent before and after the CTX-SBT treatment of the newborn.

The blood type of the mother was O and RhD-positive, and the titers of plasma IgG anti-A and anti-B were both in the range 4,096–8,192, while DAT for anti-IgG and anti-C3d were negative. The newborn’s blood type was B and RhD-positive. No irregular RBC antibodies or drug-dependent antibodies related to CTX or SBT was detected in the plasma, the acid eluent of the blood samples taken before and after the newborn’s CTX-SBT treatment, or in the plasma of the mother. Before the newborn received CTX-SBT treatment, the results of DAT for anti-IgG and anti-C3d were negative and anti-B was positive (2+) in both plasma and acid eluent. After the newborn received CTX-SBT treatment, the results of DAT for anti-IgG (3+) and anti-C3d (2+) were positive, and anti-B was not detected in plasma, while stronger anti-B (3+) was detected in acid eluent. The details of all results of the newborn’s immunohematology tests are shown in **Table 1** and **Figure 1**.

### *In Vitro* Verification of NIPA Related to CTX Versus SBT

To further validate the mechanism of hemolysis, the WRBCs and the sodium citrate anticoagulated plasma from the newborn before CTX-SBT treatment were incubated with SBT at final concentrations of 10, 5, 3 and 1mg/mL at 37°C for 3 h, and then DAT was performed. The results of DAT showed that the anti-IgG levels were 4+, 3+, 2+, 2+, and anti-C3d were 2+, 2+, 1+, and 1+, respectively. However, when the plasma of the newborn was replaced with other sodium citrate anticoagulated AB type plasma, the DAT for anti-IgG was positive while that for anti-C3d was negative. When these plasma or SBT solution was replaced by phosphate buffer solution (PBS), or the SBT was replaced by a final concentration of 10mg/mL CTX, DAT of both for anti-IgG and for anti-C3d were negative. The details of all test results are shown in **Table 2**.

**Table 2:** *In vitro* verification experiment of NIPA related to CTX versus SBT.

NO.	Reactive materials									Incubation conditions	Direct antiglobulin test	
	Newborn	Newborn	AB	PBS	100mg/mL	20mg/mL	100mg/mL	20mg/mL	Drug final concentration (mg/ml)		anti-IgG	anti-C3d
	RBCs	plasma	plasma		SBT	SBT	CTX	CTX-SBT				
	(μl)	(μl)	(μl)	(μl)	(μl)	(μl)	(μl)	(μl)				
1	20	160	/	/	20	/	/	/	10	37°C,3h	4+	2+
2	20	170	/	/	10	/	/	/	5	37°C,3h	3+	2+
3	20	150	/	/	/	30	/	/	3	37°C,3h	2+	1+
4	20	170	/	/	/	10	/	/	1	37°C,3h	2+	1+
5	20	160	/	/	/	/	/	20	2	37°C,3h	2+	1+
6	20	/	160	/	20	/	/	/	10	37°C,3h	4+	Negative
7	20	/	/	160	20	/	/	/	10	37°C,3h	Negative	Negative
8	20	160	/	20	/	/	/	/	0	37°C,3h	Negative	Negative
9	20	160	/	/	/	/	20	/	10	37°C,3h	Negative	Negative

Newborn RBCs: washed and packed red blood cells prepared by the newborn’s blood samples collected before the CTX-SBT treatment. Newborn plasma: plasma isolated from the blood samples that were anticoagulated with sodium citrate and collected before the CTX-SBT treatment of the newborn. AB plasma: sodium citrate anticoagulated AB type healthy human plasma with negative irregular red blood cell antibodies. PBS, phosphate buffer solution with pH 7.3. SBT, sulbactam; CTX, cefotaxime; +, strong.

## Discussion

The etiology of HDN is the incompatibility of mother–infant RBC blood groups, and the maternal IgG RBC antibodies are actively transported to the fetus through the placenta and bind to fetal (newborn) RBC blood group antigens. The RBCs bound to IgG antibodies are destroyed by macrophages mainly in the spleen and other reticuloendothelial tissues, which belongs to extravascular hemolysis. The main clinical manifestations of HDN are anemia and hyperbilirubinemia (18, 19, 24). The allo-immune blood group antibodies that can cause severe HDN mainly include anti-D, anti-c and anti-E of the Rh blood group system (18, 19, 24), anti-K of the Kell blood group system (24–27), anti-Di^a^ and anti-Di^b^ of the Diego blood group system (28, 29), anti-M and anti-Mur of the MNSs blood group system (30–33), etc. While, HDN caused by ABO blood group antibodies is the most common among Asian populations, and the incidence and severity are much higher than those of Caucasian populations. The reason for the significant differences in incidence and severity of ABO-HDN in different populations is still unclear (34–37). It may be that the ABO blood group antigen has not matured during the fetal period and has low reactivity with incompatible antibodies from the mother. The vast majority of newborns with ABO-HDN are mild cases, there are no reports of aborted due to fetal hemolysis caused by maternal-fetal ABO blood group incompatibility. However, in Asia, newborn hyperbilirubinemia caused by ABO-HDN is relatively common, and a few cases of bilirubin encephalopathy may occur, or the newborns must be treated with exchange blood transfusion (20, 22).

In this case, the mother’s blood type was O and RhD-positive, and her plasma had a high titer of IgG anti-A and IgG anti-B (both 4,096 – 8,192). The newborn’s blood type was B and RhD-positive, and his clinical manifestations and immunohematology test results were consistent with the diagnostic criteria of ABO-HDN (anti-B in both plasma and acid eluent were 2+) before the CTX-SBT treatment. However, ABO-HDN can cause acute and intravascular hemolysis in rare cases (38, 39). After treatment with CTX-SBT, the newborn developed hypoxia, metabolic acidosis, dark red plasma, dark brown urine, rapid decrease of hemoglobin (Hb), rapid increase of glutamic-oxalacetic transaminase (GOT) and lactate dehydrogenase (LDH), positive DAT for anti-IgG and anti-C3d, which were consistent with the characteristics of DIIHA related acute intravascular hemolysis.

Causes of DIIHA can be classified into two broad categories: drug-induced antibodies and NIPA (3, 4, 23). In this case, severe intravascular hemolysis occurred after the first CTX-SBT treatment at 43 h after birth, and no drug-dependent antibodies related to CTX or SBT were detected in the blood of the mother or the newborn, which would exclude DIIHA caused by drug-dependent antibodies related to CTX and SBT. Thus the acute intravascular hemolysis in the newborn was probably caused by the NIPA mechanism of CTX-SBT.

Characteristics of a NIPA-mediated DIIHA include: (1) the drug causes non-specific binding of the plasma IgG to the erythrocyte membrane, it also causes complement binding to the erythrocyte membrane, giving a positive DAT result for high-titer IgG with or without C3, (2) drug-induced antibodies are negative both in the patient’s serum and in an RBC eluent, (3) after incubation with the drug, high titers of IgG and C3 reactivity with drug-coated RBCs are observed, the proteins can be detected by the antiglobulin test, and (4) extravascular hemolysis (3, 23, 40). Early observation has shown that NIPA patients treated with cefothiophene showed positive results in an erythrocyte DAT using an antibody reagent against albumin, complement, fibrinogen and immunoglobulin, but the patients did not show hemolytic anemia (3). Later, NIPA induced by ampicillin-sulbactam sodium compound (unasyn), termetin-clavulanate compound, tazobactam and other drugs containing beta-lactamase inhibitors was observed, marked by DAT-positive erythrocytes and hemolytic anemia. Monocyte monolayer assay (an *in vitro* test for predicting the survival rate of erythrocytes *in vivo*) also revealed that the survival time of erythrocytes was shortened. It is suggested that the high level of plasma IgG may be related to the hemolytic anemia caused by NIPA which is induced by beta-lactamase inhibitors (2, 41–43). However, to date there are no reports of NIPA induced by CTX.

**Figure 2:**
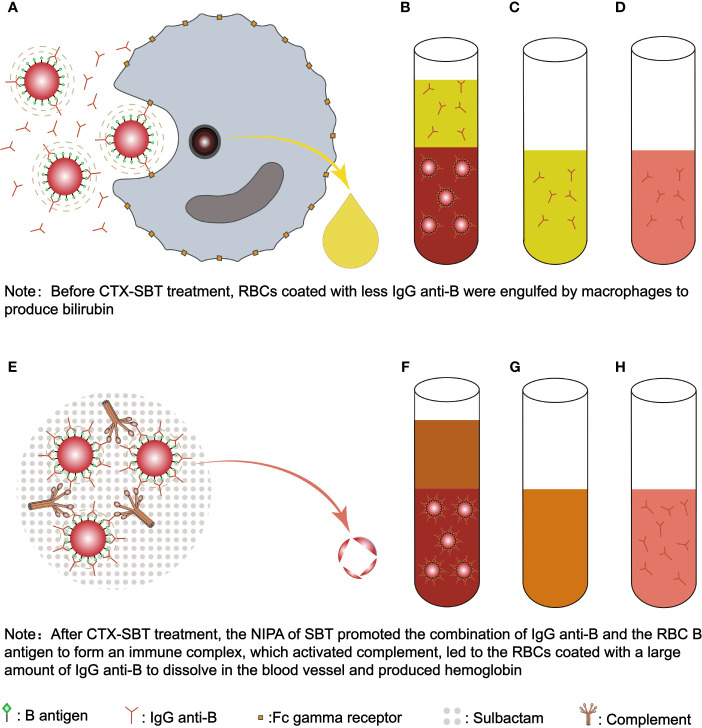
The mechanism of SBT’s NIPA induced acute intravascular hemolysis in the newborn with ABO-HDN. Before the newborn received CTX-SBT treatment, RBCs coated with less IgG anti-B were engulfed by macrophages to produce bilirubin **(A)**. Since IgG anti-B were present in both plasma and RBCs **(B)**, the anti-globulin method was used to detect IgG anti-B in plasma **(C)** and RBC acid eluent **(D)**, the results were both positive (2+). Because the affinity between the newborn’s B antigen and IgG anti-B was low, and IgG anti-B bound to RBCs were less, it was not enough to activate complement, so the results of DAT for anti-IgG and anti-C3d were negative. After the newborn received CTX-SBT treatment, the NIPA of SBT promoted the combination of maternal IgG anti-B and the newborn’s RBC B antigen to form an immune complex, which activated complement, led to the RBCs coated with a large amount of IgG anti-B to dissolve in the blood vessel and produced hemoglobin **(E)**. Since the NIPA promoted almost all maternal IgG anti-B in plasma to bind to the newborn’s RBCs **(F)**, IgG anti-B cannot be detected in plasma **(G)**, while stronger IgG anti-B were detected in acid eluent **(H)**. Since enough IgG anti-B and C3d were bound to the newborn’s RBCs, the results of DAT for anti-IgG (3+) and anti-C3d (2+) were positive. FcγRn, Fc gamma receptor; SBT, sulbactam; CTX-SBT, cefotaxime sodium - sulbactam sodium combination; RBC, red blood cell; RBCs, red blood cells; NIPA, non-immunologic protein adsorption; DAT, direct antiglobulin test; ABO-HDN, ABO blood type incompatibility hemolytic disease of newborn.

In this case, anti-B levels in plasma and acid eluent were both 2+, while DAT for anti-IgG and anti-C3d were both negative before CTX-SBT treatment. However after CTX-SBT treatment, DAT for anti-IgG (3+) and anti-C3d (2+) were positive, while anti-B was not detected in plasma, and a stronger anti-B (3+) result was obtained in the acid eluent compared to the acid eluent before CTX-SBT treatment. These data suggest that CTX-SBT can induce NIPA, but the shortened survival time of erythrocytes induced by NIPA cannot explain the acute intravascular hemolysis in the newborn after CTX-SBT treatment. Because the erythrocytes of the newborn expressed B antigen, the NIPA of CTX-SBT promoted the specific binding of anti-B immunoglobulin to the B antigen of the erythrocyte membrane, forming an immune complex, and then activated complement, which caused acute intravascular hemolysis. Our *in vitro* test verification experiments showed that CTX had no NIPA effect, while the therapeutic concentrations of SBT had a strong NIPA effect. Therefore, we concluded that the NIPA of this newborn after receiving CTX-SBT was caused by the SBT in CTX-SBT. Serum contains abundant and active complement, which is more suitable for *in vitro* tests to verify that the NIPA effect of SBT promotes the combination of specific antibodies with erythrocyte antigens to form immune complexes, and then activates complement. However, the amount of serum samples collected before the newborn received CTX-SBT treatment could not meet the needs of the trial we designed. Other studies have shown that complement in plasma is not completely inhibited, but partially inhibited, and incubation at 37°C is beneficial to complement activation in plasma (44). Therefore, we used the sodium citrate anticoagulant plasma of the newborn collected before CTX-SBT treatment incubated with the newborn’s own RBCs and SBT solution at 37°C to verify the NIPA effect of SBT. The mechanism of SBT’s NIPA effect promotes the specific binding of maternal IgG anti-B in newborn’s plasma with newborn’s RBC B antigen to form an immune complex, which then activates complement and causes acute intravascular hemolysis is shown in **Figure 2**.

Meanwhile, the number of platelets in the newborn’s blood decreased from 321.00×10^9^/L to 85.00×10^9^/L within 5 h after receiving CTX-SBT. Necrotizing enterocolitis (infection) may be one of the causes of thrombocytopenia (45). Moreover, more than 100 drugs have been proven to cause drug-induced immune thrombocytopenia (DIIT) (46). The mechanism of DIIT is similar to that of DIIHA, being mainly caused by drug-induced antibodies, hapten-dependent antibodies and drug-induced platelet-reactive autoantibodies produced by drugs (46–50). However, for the same reasons that we excluded the possibility that the DIIHA was associated with drug-based antibodies, in this case we also ruled out that the DIIT caused by drug-induced antibodies.

However, other studies have shown that weakly-reactive platelet antibodies can significantly increase the affinity for platelet glycoprotein epitopes through the bridging of drugs and platelets, leading to platelet destruction (50, 51). Ueda et al. reported a case of severe fetal and neonatal alloimmune thrombocytopenia (FNAIT) caused by high-titer anti-A (titers of 2,048–4,096) of pregnant women entering into the fetus, confirming that maternal and newborn incompatibility of ABO blood group antibodies can destroy neonatal platelets (52). The NIPA effect of SBT also has the potential to promote further binding of plasma proteins (including blood group antibodies) to platelets. In this case, the newborn’s plasma contained IgG anti-B from the mother, while his blood type was type B, and the platelet membrane expressed the B antigen. Therefore, anti-B in the plasma may be enhanced the specific binding to platelet membrane B antigen through the bridging effect of drugs and the effect of NIPA, thereby causing platelet destruction.

CTX and other β-lactam antibiotics combined with SBT are widely used in the anti-infective treatment of neonatal patients. However, the results of this case study indicate that when newborns have antibodies derived from the mother that are incompatible with their own blood type, SBT may cause acute intravascular hemolysis of the newborn through the NIPA mechanism. While CTX has no NIPA effect and will not promote the formation of immune complexes between maternal blood group antibodies and neonatal RBC corresponding blood group antigens in newborns with maternal-infant blood group incompatibility, and only administration of β-lactam antibiotics such as CTX will not cause acute intravascular hemolysis in newborns with HDN. It suggests that it is necessary to screen a list of drugs suitable for newborns with incompatible mother–infant blood type to avoid aggravating hemolysis due to the NIPA mechanism of the drugs. In addition to SBT, drugs known to induce NIPA include cefotetan, cephaloridine, cephalotinin, cisplatin, clavulanic acid, diglycoaldehyde, oxaliplatin, suramin, and tazobactam (4). We recommend that these drugs should not be administered to newborns with maternal-infant blood group incompatibility.

## Data Availability Statement

The raw data supporting the conclusions of this article will be made available by the authors, without undue reservation.

## Ethics Statement

This study was authorized by the Ethics Committee of Dongguan Maternal and Child Health Care Hospital. Written informed consent to participate in this study was provided by the participants’ legal guardian/next of kin. Written informed consent was obtained from the individual(s) for the publication of any potentially data included in this article.

## Author Contributions

YJW, YW, and YY collected samples and clinical data and performed clinical diagnosis. YJW, YW, BCC, JQL and GPG performed laboratory analysis, and YJW and FX designed the study, analyzed and interpreted the data, and wrote the paper. All authors contributed to the article and approved the submitted version.

## Funding

This work was supported mainly by a grant from the Social Science and Technology Development Key Project of Dongguan City (201950715007214) and the National Natural Science Foundation of China (31970558, 32170617), and Natural Science Foundation of Guangdong Province (2020B1515120009).

## Conflict of Interest

The authors declare that the research was conducted in the absence of any commercial or financial relationships that could be construed as a potential conflict of interest.

## Publisher’s Note

All claims expressed in this article are solely those of the authors and do not necessarily represent those of their affiliated organizations, or those of the publisher, the editors and the reviewers. Any product that may be evaluated in this article, or claim that may be made by its manufacturer, is not guaranteed or endorsed by the publisher.

## Abbreviations

HDN, hemolytic disease of newborn; ABO-HDN, ABO blood type incompatibility hemolytic disease of newborn; DIIHA, drug-induced immune haemolytic anaemia; NIPA, non-immunologic protein adsorption; RBC, red blood cell; RBCs, red blood cells; WRBCs, washed red blood cells; DAT, direct antiglobulin test; CTX, cefotaxime; SBT, sulbactam; CTX-SBT, cefotaxime sodium - sulbactam sodium combination; PBS, phosphate buffer solution; Hb, hemoglobin; GOT, glutamic-oxalacetic transaminase; LDH, lactate dehydrogenase; DIIT, drug-induced immune thrombocytopenia.
