# Supplementary material for: Case Report: First Case of Cefotaxime-Sulbactam-Induced Acute Intravascular Hemolysis in a Newborn With ABO Blood Type Incompatibility by the Mechanism of Non-Immunologic Protein Adsorption
Source: Front Immunol. 2021 Dec 22;12:698541. doi: 10.3389/fimmu.2021.698541 (PMC8727536; doi:10.3389/fimmu.2021.698541)
Supplement: Supplementary file 1 [file DataSheet_1.doc]

**Methods**

**Red blood cell type identification and antibody testing**

The newborn's blood samples were collected at 32 and 43 h after birth (before he was given CTX-SBT treatment) and 48 h after birth (after administration of CTX-SBT), and samples of his mother's blood were also taken for identification of ABO and RhD blood groups. The micro-column gel anti-IgG method was used to detect IgG ABO blood group antibodies, and to screen for erythrocyte irregular antibody. For the AB fresh frozen plasma infused to the newborn, the saline medium method was used to detect the ABO antibody, and the micro-column gel anti-IgG method was used to detect the erythrocyte irregular antibody.

**Direct antiglobulin test**

Direct antiglobulin test (DAT) including anti-IgG and anti-C3d was conducted on samples from the newborn and his mother according to the previous description^[1]^. The tested samples include the newborn's blood samples were collected at various time points before (32 h and 43 h after birth) and after (48 h after birth) the CTX-SBT treatment and his mother's blood sample.

**Erythrocyte elution test and erythrocyte antibody detection in eluent**

The red blood cells (RBCs) were separated from the newborn's blood samples collected at 32, 43 and 48 h after birth. The erythrocyte elution was performed at 56℃. Then, the micro-column gel anti-IgG method was used to detect the ABO antibody and erythrocyte irregular antibody in the erythrocyte eluent.

**Drug-induced antibody testing**

Drug-induced antibody testing was performed as described previously^[2]^, to detect drug-dependent antibodies against CTX and SBT in the plasma and the erythrocyte eluent of blood samples from the newborn before and after CTX-SBT treatment, and in his mother's plasma. Briefly, 40 mg/mL CTX solution and 40 mg/mL SBT solution were prepared at pH 7.3 in phosphate-buffered saline (PBS). Washed red blood cells (RBCs) were prepared from DAT-negative O-type healthy human blood samples. CTX-coated RBCs and SBT-coated RBCs were prepared by suspending washed RBCs at a concentration of 10% in a solution of either 40 mg/mL CTX or 40 mg/mL SBT, and then incubating at 37℃ for 1 h, after which they were washed 4 times with pH 7.3 PBS. In addition, 1 mg/mL CTX solution and 1 mg/mL SBT solution were prepared in pH 7.3 PBS. The methods of "testing drug-induced antibodies with drug-treated RBCs" and "testing drug-induced antibodies in the presence of a drug solution" were employed to detect drug-dependent antibodies against CTX and SBT as described by Leger RM et al^[2]^.

**Controlled trial of nipa induced by CTX versus SBT *in vitro***

The anticoagulant-treated blood sample collected from the newborn before CTX-SBT treatment was centrifuged, and the plasma was separated and red blood cells were packed. The packed red blood cells were washed three times with PBS (pH 7.3), then mixed with the newborn plasma or PBS, or with AB type healthy human plasma, and then incubated with different final concentrations of SBT or CTX at 37°C for 3 h. Finally, the micro-column gel anti-IgG and monoclonal anti-C3d were used to perform DAT for the RBCs in each tube.

Reference

1.Parker, V. & Tormey, C.A. The Direct Antiglobulin Test: Indications, Interpretation, and Pitfalls. *Arch. Pathol. Lab. Med*. **141**, 305-310 (2017).

2.Leger, R.M., Arndt, P.A., & Garratty, G. How we investigate drug-induced immune hemolytic anemia. *Immunohematology* **30**, 85-94 (2014).
